# Peritumoral adipose tissue promotes lipolysis and white adipocytes browning by paracrine action

**DOI:** 10.3389/fendo.2023.1144016

**Published:** 2023-04-26

**Authors:** Priscila Pagnotta, Mariana Gantov, Sabrina Fletcher, Antonella Lombardi, María Lujan Crosbie, Natalia Santiso, Anabela Ursino, Celeste Frascarolli, Alicia Amato, Rubén Dreszman, Juan Carlos Calvo, Judith Toneatto

**Affiliations:** ^1^ Institute of Biology and Experimental Medicine (IBYME), CONICET, Buenos Aires, Argentina; ^2^ Department of Biological Chemistry, Faculty of Exact and Natural Sciences, University of Buenos Aires, Buenos Aires, Argentina; ^3^ Institute of Neurosciences (INEU) FLENI-CONICET, Buenos Aires, Argentina; ^4^ Breast Surgery Section, Churruca-Visca Police Medical Centre, Buenos Aires, Argentina; ^5^ Microsurgery Clinic, Buenos Aires, Argentina

**Keywords:** human breast adipose tissue, paracrine action, browning, lipolysis, tumor microenvironment

## Abstract

**Background:**

Stromal adipocytes and tumor breast epithelial cells undergo a mutual metabolic adaptation within tumor microenvironment. Therefore, browning and lipolysis occur in cancer associated adipocytes (CAA). However, the paracrine effects of CAA on lipid metabolism and microenvironment remodeling remain poorly understood.

**Methods:**

To analyze these changes, we evaluated the effects of factors in conditioned media (CM) derived from explants of human breast adipose tissue from tumor (hATT) or normal (hATN) on morphology, degree of browning, the levels of adiposity, maturity, and lipolytic-related markers in 3T3-L1 white adipocytes by Western blot, indirect immunofluorescence and lipolytic assay. We analyzed subcellular localization of UCP1, perilipin 1 (Plin1), HSL and ATGL in adipocytes incubated with different CM by indirect immunofluorescence. Additionally, we evaluated changes in adipocyte intracellular signal pathways.

**Results:**

We found that adipocytes incubated with hATT-CM displayed characteristics that morphologically resembled beige/brown adipocytes with smaller cell size and higher number of small and micro lipid droplets (LDs), with less triglyceride content. Both, hATT-CM and hATN-CM, increased Pref-1, C/EBPβ LIP/LAP ratio, PPARγ, and caveolin 1 expression in white adipocytes. UCP1, PGC1α and TOMM20 increased only in adipocytes that were treated with hATT-CM. Also, hATT-CM increased the levels of Plin1 and HSL, while decreased ATGL. hATT-CM modified the subcellular localization of the lipolytic markers, favoring their relative content around micro-LDs and induced Plin1 segregation. Furthermore, the levels of p-HSL, p-ERK and p-AKT increased in white adipocytes after incubation with hATT-CM.

**Conclusions:**

In summary, these findings allow us to conclude that adipocytes attached to the tumor could induce white adipocyte browning and increase lipolysis as a means for endocrine/paracrine signaling. Thus, adipocytes from the tumor microenvironment exhibit an activated phenotype that could have been induced not only by secreted soluble factors from tumor cells but also by paracrine action from other adipocytes present in this microenvironment, suggesting a “domino effect”.

## Introduction

Over the past decade, tumors have been described as complex organs, surpassing even the complexity of healthy tissues. For this reason, cancer and tumorigenesis must be studied by analyzing both tumor cells and its microenvironment (1). Tumor microenvironment (TME) comprises different cell types as well as components of the extracellular matrix. These components can positively or negatively regulate the progression of the disease. In this sense, normal cells of the tumor microenvironment are not passive components; they can be recruited by tumor cells and actively participate in key cancer processes (1, 2).

Adipose tissue (AT) is the main breast stromal component and its role in tumor progression has become the focus of study in recent years (3–9).

Adipogenesis is a complex process controlled by multiple genes and with a highly regulated expression. In mammals, there are early regulators of adipogenesis: the peroxisome proliferator-activated receptor γ (PPARγ), and the CCAAT/enhancer binding proteins (C/EBPα, β and δ) while adiponectin, leptin, fatty acid binding protein 4 (FABP4), perilipin 1 (Plin1), among others, act later in the process and are responsible for the formation of mature adipocytes (10). Importantly, to maintain mature AT, PPARγ expression/activation is required. Contrarily, Pref-1 is a protein which maintains the undifferentiated state of preadipocyte by preventing adipogenesis. This occurs due to activation of the ERK1/2 pathway, which downregulates the expression of adipocyte markers and secreted factors (10, 11).White adipocytes can undergo browning. The browning process involves a shift in adipocyte phenotype (white to beige). Beige adipocytes from white AT (WAT) are identified by their genotypic and phenotypic characteristics, such as the expression of PRDM16 (proline rich domain containing 16), PGC1α (peroxisome proliferator activated receptor γ coactivator 1 α), UCP1 (uncoupling protein 1) and TBX1 (TATA box 1), small lipid droplets (LDs) and a high number of mitochondria, necessary for their thermogenic activity (12).

Adipocytes closest to tumor cells, generally present on the invasive front of carcinomas, exhibit an altered phenotype characterized by smaller size, loss of adipocyte-associated markers and intracellular loss of lipids (3, 13). They have a different secretion profile compared to other adipocytes. Adipocytes close to tumor cells, but not adjacent to the invasive front, called cancer-associated adipocytes (CAAs), were found to increase the expression of proteases, adipokines, and proinflammatory cytokines (14–16). Recently, a possible browning process of adipocytes within tumor microenvironment has been postulated. Evidence of this were phenotypic modifications of peritumoral AT, and changes in the expression of in beige/brown adipose-related markers (17–19). CAAs undergo a metabolic reprogramming of almost all macronutrients, such as carbohydrates, lipids, and amino acids, promoting tumor progression (20). Cancer cells release signaling molecules, including TNF-α and IL-6 that reprogram lipid metabolic pathways in CAAs surrounding tumors and in distal locations to trigger local and systemic lipolysis, causing adipose atrophy and cachexia in cancer patients (21). In addition, there is evidence that AT sets in motion adaptation mechanisms leading to oxidative stress because of the presence of tumor cells, such as lactate release, which favors an oxidative phenotype with uncoupled mitochondria with increased expression of UCP1 and high oxidation rate (22, 23). Several lines of evidence support the existence of a close relationship between the lipolysis process and UCP1 activation: 1- thermogenesis is activated by agents that increase lipolysis (24) and, 2- thermogenesis is affected in knockout mice for triglyceride lipase (ATGL), inducing deficient triacylglycerol (TG) hydrolysis in adipocytes (25–27) or expression of a mutant Plin1, a PAT protein that coats LDs that cannot phosphorylate and, therefore, does not allow the access of hormone-sensitive lipase (HSL) to LDs in order to hydrolyze TG (28).

Wu et al. proposed that breast cancer tumor cells promote browning and lipolysis in adipocytes as one of the first steps in adipocyte-tumor crosstalk (29). Recent studies have revealed the first indications that AT associated with renal cancer undergoes phenotypic and gene expression modifications, that suggest an increase in lipolysis and the browning of peritumoral AT (30). We have recently demonstrated the beige adipocytes-epithelial cells crosstalk and how beige adipocytes participate and modulate breast cancer tumor progression, in a murine model (31). Our group has previously demonstrated that peritumoral adipocytes display a less differentiated state than adipocytes from a normal microenvironment (13). In fact, the conditioned media (CM) derived from explants of human breast adipose tissue from tumors (hATT) improve proliferation and migration of breast cancer epithelial cell lines, in contrast to CM from explants of human adipose tissue from normal breasts (hATN) (13, 32). These findings are in accordance with the differential components present in hATT- and hATN-CM. hATT-CM showed greater protein abundance and diversity, related to the immune system, lipid metabolism, complement activity, and proteins involved in signal transduction and cell communication. Also, we showed that IL-6, monocyte chemotactic protein-2 (MCP-2) and GRO cytokines were necessary and sufficient to differentiate hATT-CM from hATN-CM, using a multivariate discriminant analysis of cytokines detected by array (33).

In this context, we hypothesize that the peritumoral AT adipocytes, modified by the presence of tumor cells, secrete factors into the medium that could change their microenvironment by paracrine action on adjacent adipocytes, inducing white adipocyte browning and lipolysis. To corroborate this hypothesis, we aimed to determine whether soluble factors from hATT can induce browning and lipid metabolism changes in mature 3T3-L1 adipocytes. Thus, we compared the size and number of LDs, size of adipocytes, TG content, glycerol release, changes in the expression levels of beige/brown markers (PRDM16, PGC1α, UCP1 and TBX1), adipogenic markers (Pref-1, C/EBPβ, PPARγ), FABP4, caveolin 1 (CAV-1) and lipolytic proteins (Plin1, HSL and ATGL). We also analyzed the changes in subcellular localization of UCP1, Plin1, HSL and ATGL, in mature 3T3-L1 white adipocytes incubated with hATT-CM *vs*. hATN-CM. To elucidate the intracellular mechanisms involved in the lipolytic process, we included the analysis of alterations in the phosphorylation state of HSL, PKA, ERK and AKT. The effect of hATT- and hATN-CM was analyzed after a short (24 h), medium (72 h) and/or long (120 h) incubation period.

This experimental approach suggests that adipocytes attached to the tumor could induce white adipocyte browning and lipolysis in its microenvironment, for paracrine signaling. This remodeling suggests a loss of normal functions in mature adipocytes attached to the tumor, acquiring others that could favor tumor progression.

## Materials and methods

### Reagents

Reagents were obtained from Sigma-Aldrich/Merck Chemical Co (St. Louis, MO, USA); multi-well plates and dishes were purchased from Jet biofilm (Biotech, CABA, Argentina); supplements and culture media were from Gibco BRL (Carlsbad, CA, USA).

### Sample collection and handling

We used explants of human breast AT from tumoral (hATT, *n* = 26) and normal (hATN, *n* = 18). hATT tissue samples were obtained from infiltrating ductal carcinomas, estrogen and/or progesterone receptor positive, on stages GH1 or GH2. No patients received prior chemotherapy or radiotherapy treatment. hATN tissue samples were collected from surgeries performed for esthetic (breast reduction) reasons. Samples were handled as previously described (32). In brief, adipose tissue (AT) samples were immersed in phosphate-buffered saline (PBS) containing gentamicin (50 µg/ml) and processed under sterile laminar flow conditions. Typically, an average of 2 hours elapsed from the time of surgical sample acquisition until processing. All patients gave their written informed consent. Samples (tumor and normal) were obtained following the approval of both IBYME (CE 025) and Churruca-Visca Police Medical Center IRBs.

### Collection and preparation of conditioned media (CM)

AT samples were obtained as previously reported (13). In summary, human adipose tissue-derived stromal cells (hATN or hATT) were cultured with M199 medium (Invitrogen™) at a ratio of 1 g tissue to 10 ml M199 and incubated for 1 h at 37°C in a 5% CO_2_ atmosphere. The medium was then replaced with fresh medium, and the tissues were incubated for 24 h. The resulting supernatant was collected and subjected to centrifugation (3 min at 400 x g) to remove cells, followed by filtration using 0.22 μm filters. The resulting supernatants were aliquoted into 1 ml fractions and immediately stored at -80°C. Control conditioned media (Ctrol-CM) was obtained by collecting serum-free M199 medium after 24 h of incubation in a culture plate at 37°C in a 5% CO_2_ atmosphere. Collected CM were diluted 1:1 in D-MEM/F-12 medium (Invitrogen, UK) with 2% bovine serum albumin [(BSA, Sigma-Aldrich/Merck), 1% BSA as final concentration] and adipocytes were treated with the diluted CM. For the experiments, we used equal volumes of hATN- and hATT-CM.

### Culture and differentiation of preadipocytes 3T3-L1

**Figure 1:**
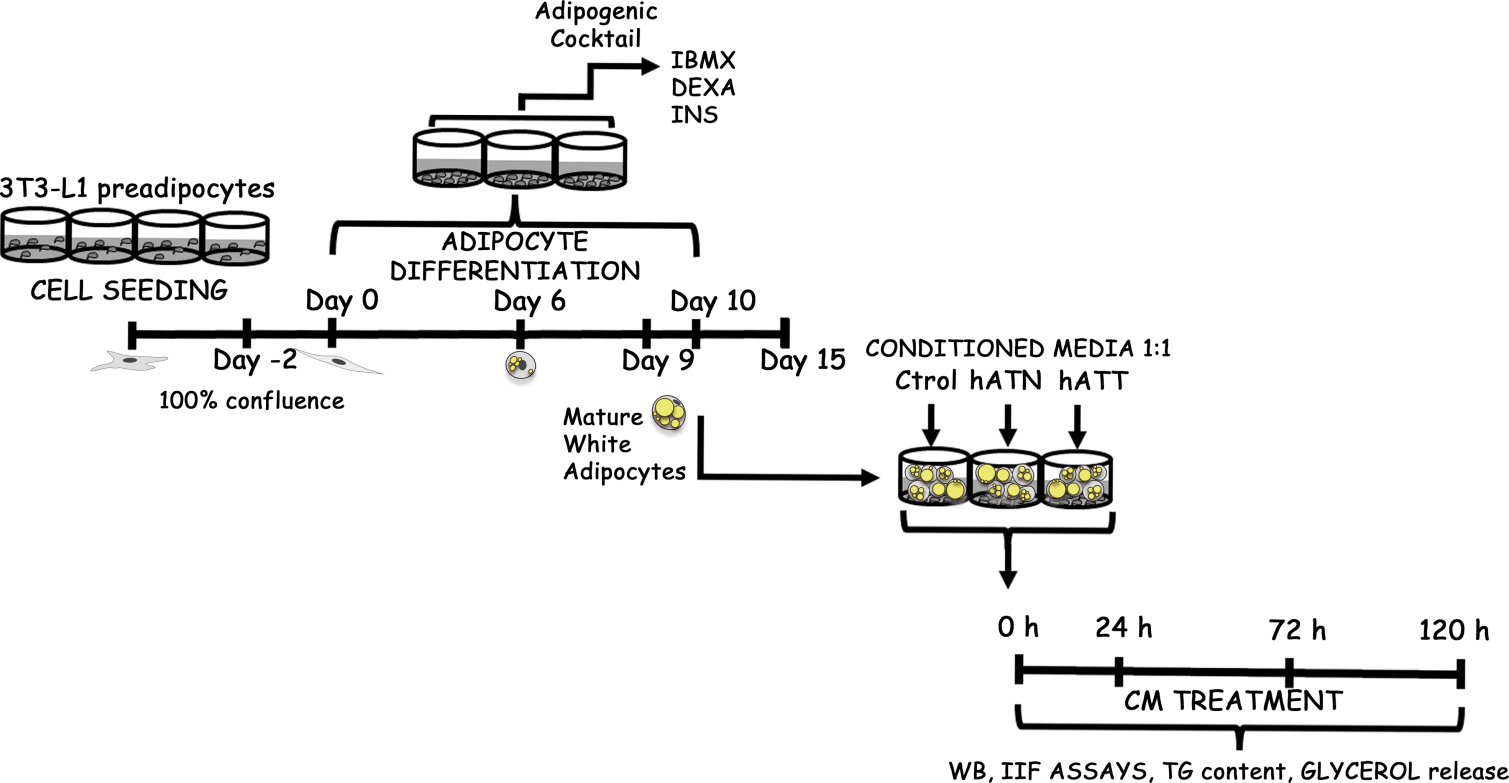
Schematic experimental approach. 3T3-L1 preadipocytes were seeded in multiwell plates and allowed to grow until they reached confluence on day 0. Two days later, an adipogenic cocktail containing 0.5 mM IBMX, 0.1 μM DEXA, and 0.1 μM rosiglitazone was added to the cells, which were then further cultured for two days. Afterward, the culture medium was replaced with complete D-MEM/F-12 containing 2 μM INS. Adipocytes were maintained in D-MEM/F-12 supplemented with 10% FBS after six days. Following nine days, a vast majority (90-100%) of the cells had differentiated into WAs that exhibited an increase in TG content and the formation of LDs, which were visualized under light microscopy either directly or following Oil Red O staining or Lipid-TOX *via* IIF. Ctrol-CM, Control conditioned media; hATN-CM, conditioned media from human normal breast adipose tissue explants; and hATT-CM, conditioned media from human breast cancer adipose tissue explants, were used. FBS, Fetal bovine serum; IIF, indirect immunofluorescence; ON, overnight; WB, Western blot; TG, triacylglycerol; IBMX, 3-isobutyl-1-methylxanthine; DEXA, dexamethasone; INS, insulin and WA, white adipocyte are the terms used in this context.

Culture and differentiation of murine 3T3-L1 preadipocyte cells (obtained from ATCC, American Type Culture Collection, USA) were performed as described previously (31). Details of the experimental approach are provided in **Figure 1**.

### Indirect immunofluorescence (IIF)

IIF assays were performed as previously reported (34). Briefly, equal number of preadipocyte 3T3-L1 cells were seeded on coverslips and differentiated into white adipocytes (WA). After 9 days, 90-100% cells had differentiated into mature 3T3-L1 adipocytes, which were treated with the different CM for 24 or 72 h. After treatment, adipocytes on coverslips were washed with PBS, then fixed for 15 min in 4% paraformaldehyde, permeabilized for 10 min with 0.5% Triton X-100 in PBS and blocked for 1 h at room temperature (RT). Adipocytes were incubated ON at 4°C with specific antibodies, and 1 h at RT with secondary antibody, previously blocked for 1 h at RT. Nuclei were counterstained with Hoechst and LDs with Lipid-TOX. Supplementary information of antibodies and dilutions used are provide in **additional file: Supplementary Table S1**. Finally, coverslips were mounted with Vectashield. Images were captured with a Spinning Disk-TIRF-Olympus DSU-IX83 microscope, using a 60x objective and then, analyzed using ImageJ software (version 1.52p; NIH). Microscope settings were the same for all samples.

### Analysis of triglyceride content

On day 9 of differentiation, 3T3-L1 adipocytes were incubated ON with serum-free D-MEM/F-12 containing 1% BSA. Subsequently, adipocytes were treated with the different CM (diluted 1:1) for the indicated times. Adipocytes were lysed with water (30 µl per well), previously washed once with PBS at RT. After 5 min of incubation, cells were scraped, and TG determined using the TG Color GPO/PAP AA Kit (Wiener Laboratorios, Rosario, Argentina).

### Measurement of glycerol release

After differentiation in 12-well plate, mature 3T3-L1 adipocytes were incubated with the different CM (diluted 1:1) for the indicated times. Then, the medium was discarded and 200 µl of phenol red-free D-MEM/F-12 containing 0.5% BSA were added, and cells incubated for 2 h. Glycerol content in media was measured using a fluorometric assay kit. This determination was performed according to the instructions from the manufacturer (Free Glycerol Assay Kit ab65337). Values were normalized to protein concentration.

### Preparation of adipocyte lysates after incubation with hATN-, hATT- or Ctrol-CM

After differentiation into 12-well plates, mature 3T3-L1 adipocytes were washed twice with PBS. Adipocytes were further incubated at 37°C for 24 h, 72 h or 120 h with diluted CM (diluted 1:1). Following incubation, adipocytes were lysed in lysis buffer (Tris-SDS, 60 mM Tris-HCl, 1% SDS, pH 6.8), pelleted by centrifugation and stored at -80°C.

### Western blot assay (WB)

Sample preparation and immunoblots were performed as previously reported (31). Pref-1, C/EBPβ LAP, C/EBPβ LIP, PPARγ, FABP4, CAV-1, UCP1, PRDM16, TBX1, PCG1α, TOMM20, Plin1, HSL, pHSL, ATGL, pPKA, ERK, pERK, AKT and pAKT were measured after incubation of white mature adipocytes with the different CM. Supplementary information of antibodies and dilutions used are provided in **Additional File: Supplementary Table S2**. Total protein in samples was quantified using DC Protein Assay kit (Bio-Rad Laboratories, Inc.). Proteins (25-50 µg) were separated in an SDS-PAGE 12% gel and transferred to polyvinylidene fluoride membranes (PVDF) (Bio-Rad Laboratories, Inc.). Membranes were later blocked with 1% BSA for 1 h (Sigma-Aldrich; Merck KGaA) and incubated with the different specific antibodies ON at 4°C. After washed, membranes were incubated with the corresponding HRP-conjugated secondary antibodies. Proteins were detected by chemiluminescence [ECL solution containing luminol (Sigma-Aldrich; Merck KGaA), p-coumaric acid (Sigma-Aldrich; Merck KGaA) and H_2_O_2_]. The ImageJ software was used to quantify the protein level. Actin levels were used as loading controls.

### Statistical analysis

Analysis was performed by one way ANOVA and multiple comparisons or Student´s t-test with Welch’s correction as appropriate, using GraphPad Prism 8 (Graphpad Software Inc.) software. The results are presented as mean ± SEM. Significance was determined according to *P<0.05, **P<0.01, ***P<0.001, and ****P<0.0001.

## Results

### Effects of soluble factors from hATT and hATN on morphology of white adipocytes

To compare soluble factors, present in hATT- and hATN-CM, that could modify adipocyte morphology, we analyzed by IIF cell size, number of LDs per cell and LDs size distribution on 3T3-L1 adipocytes incubated with the different CM (hATT-, hATN- and Ctrol-CM) for 24, 72 and 120 h.

**Figure 2:**
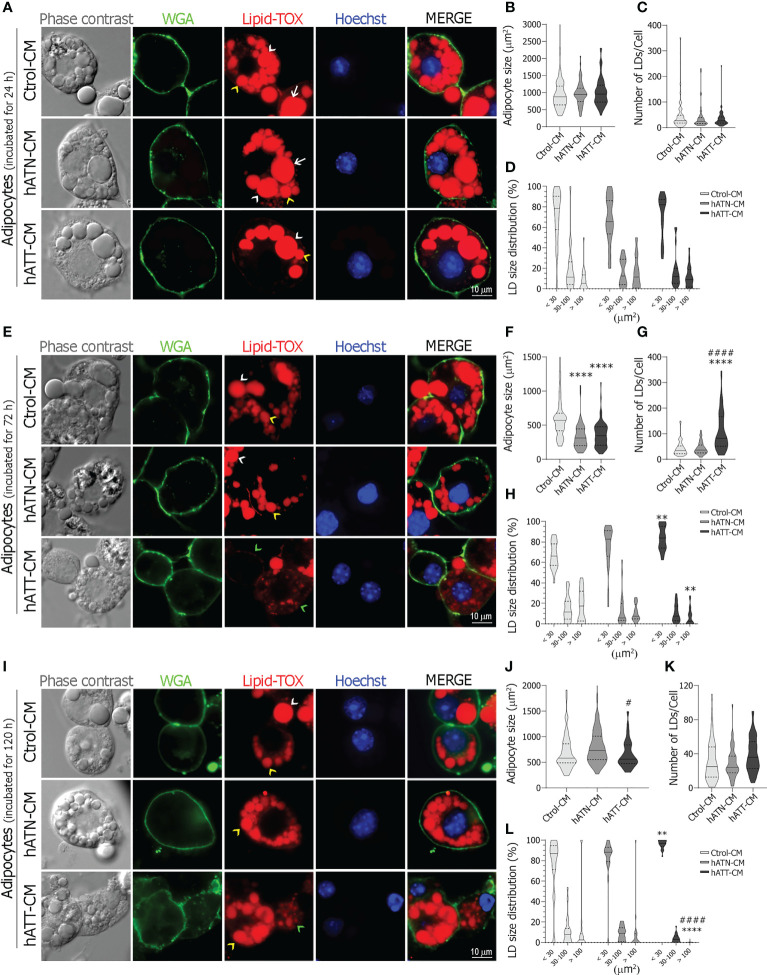
Effect of CM from hATN and hATT on morphological parameters in mature adipocytes. 3T3-L1 adipocytes were incubated with hATN-, hATT- or Ctrol-CM for 24 **(A)**, 72 **(E)** and 120 h **(I)**, subjected to IIF with the indicated antibodies, and images were analyzed by confocal microscopy (magnification x600; see Materials and Methods section for further details). White arrows, white arrow heads, yellow arrow heads and green arrow heads show examples of large, medium, small and micro-LDs, respectively. Violin plots represent the adipocyte size (μm^2^) (**B, F, J**, adipocytes incubated with the different CM for 24, 72 and 120 h, respectively), the number of LDs per cell (**C, G, K**, adipocytes incubated with different CM for 24, 72 and 120 h, respectively) and percentage of LDs according to size (micro < 10 μm^2^ and small 10 to 30 μm^2^, medium from 30 to 100 μm^2^, and large > 100 μm^2^) (**D, H, L**, adipocytes incubated with different CM for 24, 72 and 120 h, respectively) from 64 adipocytes (n = 3 experiments in duplicate). Tukey’s multiple comparison test was performed; **P<0.01, and ****P<0.0001 hATT-CM or hATN-CM *vs.* Ctrol-CM; #P<0.05, and ####P<0.0001 hATT-CM *vs.* hATN-CM. Ctrol-CM, control conditioned media; hATN-CM, conditioned media from human normal breast adipose tissue explants; hATT-CM, conditioned media from human breast cancer adipose tissue explants; LDs, Lipid droplets; WGA, Wheat germ agglutinin.

Although hATT-CM induced a morphological transition, increasing the number of small-LDs per cell in adipocytes incubated for 24 h (**Figure 2A**, *Lipid-TOX bottom panel, yellow arrow heads*), differences did not achieve statistical significance with respect to hATN- and Ctrol-CM (**Figure 2D**). No effect was observed for hATT- and hATN-CM on adipocyte size and LDs number after 24 h of incubation (**Figures 2B, C**, respectively). Contrarily, after 72 h of incubation, hATT-CM and hATN-CM significantly decreased adipocyte cell size (**Figure 2F**). Moreover, hATT-CM increased the average number of small and micro-LDs within adipocytes (**Figure 2G**) which underwent a morphological transition at the expense of a decrease in large-LDs (**Figure 2E**, *Lipid-TOX bottom panel, green arrow heads showing micro-LDs in adipocytes incubated with hATT-CM*, and **Figure 2H**). The effect of hATT-CM on LDs size was greater after 120 h of treatment (**Figures 2I, L**, hATT-CM *vs.* hATN- or Ctrol-CM). ATT-CM significantly decreased cell size in comparison to hATN-CM (**Figure 2J**). No effect was observed for hATT- and hATN-CM on LDs number after 120 h of incubation (**Figure 2K**). hATT-CM induced not only cell and LDs size changes but also morphological changes in adipocytes, which lost their characteristic round shape (**Figure 2I**, *WGA bottom panel vs. middle and top panels*) with mostly small and micro-LDs (**Figure 2L**). Additionally, we analyzed the effect of hATT- and hATN-CM on the nuclei area of white adipocytes; after 72 h of incubation, hATT-CM significantly increased nucleus size compared to Ctrol-CM (**Additional File: Supplementary Figure S1**).

**Figure 3:**
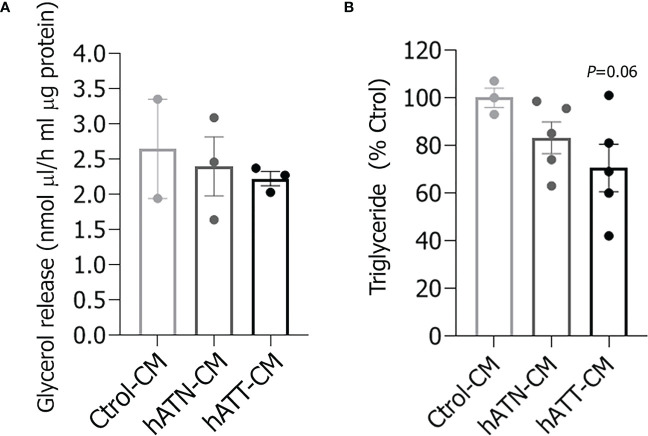
Effect of CM from hATN and hATT on triglyceride content in adipocytes and glycerol release into the medium. 3T3-L1 adipocytes were incubated with hATN-, hATT- or Ctrol-CM, and then the glycerol released into the media **(A)** and the triglyceride cell content **(B)** were determined by colorimetric assay at 505 and 570 nm, respectively (n = 3 experiments in duplicate). Student’s t-test with Welch´s correction was performed; *p* = 0.06 adipocytes incubated with hATT-CM *vs.* Ctrol-CM. Ctrol-CM, control conditioned media; hATN-CM, conditioned media from human normal breast adipose tissue explants; hATT-CM, conditioned media from human breast cancer adipose tissue explants.

To further confirm that hATT-CM promotes lipid mobilization, we determined glycerol, a product of TG hydrolysis, and TG content in adipocytes incubated for 24 and 72 h, respectively. Although glycerol released from adipocytes incubated with hATT- and hATN-CM was not significantly different, a decrease in TG content was observed after 72 h of incubation with hATT-CM with respect to hATN- and Ctrol-CM (**Figures 3A, B**, respectively).

### Effects of soluble factors from hATT and hATN on protein expression of adipogenic and mature adipose-related markers in white adipocytes

**Figure 4:**
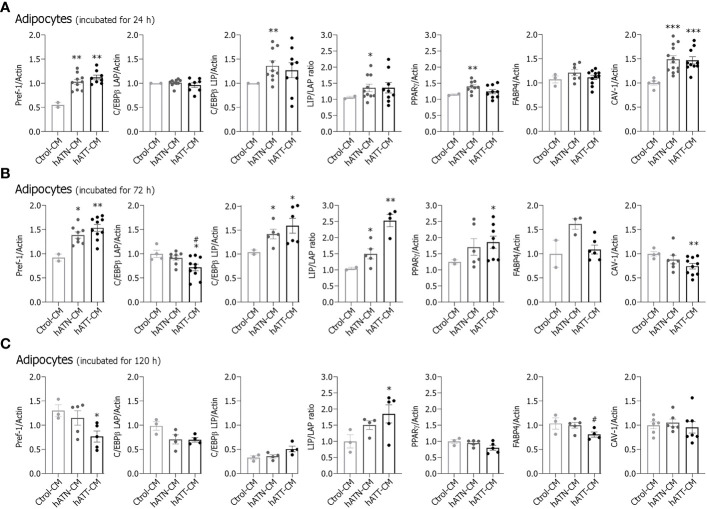
Effect of CM from hATN and hATT on the protein expression of adipogenic and mature adipocyte markers. 3T3-L1 adipocytes were incubated with hATN-, hATT- or Ctrol-CM for 24 **(A)**, 72 **(B)** and 120 h **(C)** and, then, lysed. The expression of the different proteins was measured by WB. Actin was used as a loading control. Images were analyzed by densitometry. (n = 4-5 experiments in duplicate). Student’s t-test with Welch´s correction was performed; *P<0.05, **P<0.01, and ***P<0.001, adipocytes incubated with hATT-CM or hATN-CM *vs.* Ctrol-CM; #P<0.05, adipocytes incubated with hATT-CM *vs.* hATN-CM. Ctrol-CM, control conditioned media; hATN-CM, conditioned media from human normal breast adipose tissue explants; hATT-CM, conditioned media from human breast cancer adipose tissue explants; Pref-1, preadipocyte factor 1; C/EBPβ, CCAAT/enhancer binding protein β; LAP, liver activating protein; LIP, liver inhibitory protein; PPARγ, peroxisome proliferator activated receptor γ; FABP4, fatty acid binding protein 4; CAV-1, Caveolin 1.

To evaluate the effect of hATT- and hATN-CM on protein expression of several adipogenic and mature adipose-related markers, we analyzed by WB protein expression of these markers in adipocytes after incubation for 24, 72 and 120 h (**Figures 4A–C**, respectively). hATN- and hATT-CM increased expression of Pref-1, a protein highly expressed in preadipocytes, in adipocytes incubated for 24 h compared to Ctrol-CM (**Figure 4A**). hATN-CM increased C/EBPβ-LIP and PPARγ expression compared to hATT- and Ctrol-CM. C/EBPβ-LAP and FABP4 expression showed no significant differences. Importantly, the expression of CAV-1, an important protein necessary for LDs formation and breakdown, was significantly increased in adipocytes incubated with hATN- and hATT-CM compared to Ctrol-CM (**Figure 4A**).

Then, we analyzed the effect of CM on adipogenic and mature adipocyte-related markers after adipocyte incubation for 72 h. hATN- and hATT-CM increased Pref-1 expression compared to Ctrol-CM, while C/EBPβ-LAP expression decreased following incubation with hATT-CM compared to hATN- and Ctrol-CM (**Figure 4B**). Contrarily, the expression levels of C/EBPβ-LIP and LIP/LAP ratio increased in after incubation with hATT- or hATN-CMs compared to Ctrol-CM. hATT-CM significantly increased PPARγ expression level with respect to hATN- and Ctrol-CM (**Figure 4B**). CAV-1 decreased after incubation with hATT-CM, and FABP4 expression levels showed a tendency to increase following incubation with hATN-CM (**Figure 4B**).

After incubation for 120 h, hATT-CM decreased Pref-1 and FABP4 expression (**Figure 4C**). On the contrary, expression levels of C/EBPβ-LIP and LIP/LAP ratio increased after incubation with hATT-CM when compared to hATN- and Ctrol-CM. PPARγ and CAV-1 expression showed no significant differences in adipocytes incubated for 120 h with hATN- or hATT-CM in comparison to Ctrol-CM (**Figure 4C**). The results indicate that hATN- and hATT-CM modulate the expression of adipogenic and mature adipose markers. However, while hATN-CM provide a suitable environment to maintain a mature adipose state, hATT-CM induce a less differentiated state after long term treatment. However, the increase of PPARγ, concomitantly with the morphological change induced by hATT-CM after 72 h of treatment, suggests that hATT-CM could induce changes in adipose phenotype (white to beige).

### Effects of soluble factors from hATT and hATN on UCP1 subcellular localization and beige/brown adipose-related markers protein expression in white adipocytes

**Figure 5:**
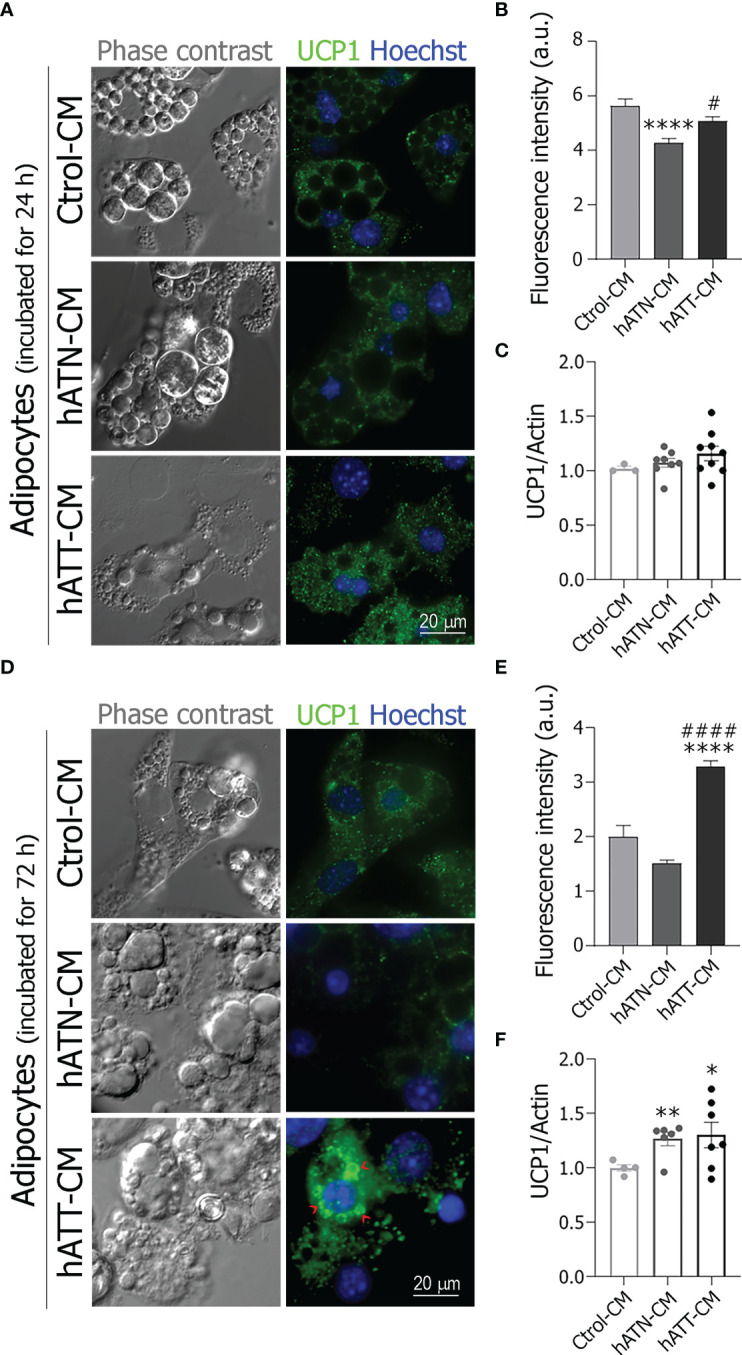
Effect of hATN- and hATT-CM on the subcellular localization and the protein expression of UCP1. 3T3-L1 adipocytes were incubated with hATN-, hATT- or Ctrol-CM for 24 **(A)** and 72 h **(D)**, and subjected to IIF with the indicated antibodies. The images were analyzed by confocal microscopy (magnification x600; see Materials and methods section for further details). Red arrow heads show examples of micro-LDs. Fluorescence intensity was measured from 43 adipocytes incubated with the different CM for 24 or 72 h (**B, E**, respectively). (n = 3 experiments in duplicate). Tukey’s multiple comparison test was performed. 3T3-L1 adipocytes were incubated with hATN-, hATT- or Ctrol-CM for 24 and 72 h, then lysed and the expression of UCP1 was measured by WB (**C, F**, respectively). Actin was used as a loading control. Images were analyzed by densitometry. (n = 3-4 experiments in duplicate). Student’s t-test with Welch’s correction was performed; *P<0.05, **P<0.01 and ****P<0.0001 adipocytes incubated with hATT-CM or hATN-CM *vs.* Ctrol-CM; #P<0.05 and ####P<0.0001 adipocytes incubated with hATT-CM *vs.* hATN-CM. a.u., arbitrary units; Ctrol-CM, control conditioned media; hATN-CM, conditioned media from human normal breast adipose tissue explants; hATT-CM, conditioned media from human breast cancer adipose tissue explants; UCP1, Uncoupling protein 1.

To evaluate whether hATT-CM promote the browning of white adipocytes, we analyzed by IIF and WB UCP1 subcellular localization and protein expression after incubation for 24 (**Figures 5A–C**) and 72 h (**Figures 5D–F**). In adipocytes incubated for 24 h with hATT- and hATN-CM, no change was observed in the punctate staining of UCP1, characteristic of its mitochondrial localization, in comparison to Ctrol-CM (**Figure 5A**, *bottom and middle panels vs. top panels*). UCP1 fluorescence intensity increased in adipocytes incubated with hATT-CM with respect to hATN-CM (**Figure 5B**). There was a tendency in UCP1 expression level to increase in adipocytes incubated with hATT-CM, though the difference was not significant (**Figure 5C**). After 72 h of incubation with hATT-CM, UCP1 was distributed around LDs, mainly micro-LDs (**Figure 5D**, *bottom right panel, red arrow heads*), with a concomitant increase in fluorescence intensity, in comparison to adipocytes incubated with hATN- or Ctrol-CM (**Figure 5D** *middle and top panels* and E). hATT-CM and hATN-CM significantly increased UCP1 protein expression after 72 h of incubation in comparison to Ctrol-CM (**Figure 5F**).

**Figure 6:**
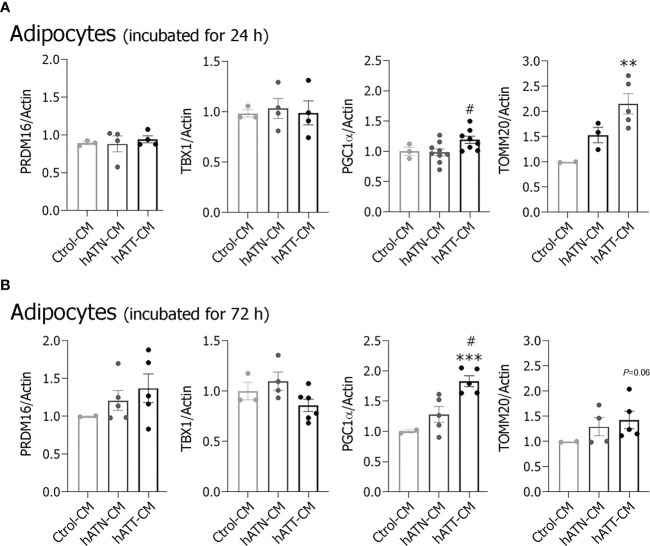
Effect of CM from hATN and hATT on the expression of beige/brown markers. 3T3-L1 adipocytes were incubated with hATN-, hATT- or Ctrol-CM for 24 and 72 h (**A, B**, respectively), and then lysed. Expression of the different proteins was measured by WB. Actin was used as a loading control. Images were analyzed by densitometry. Student’s t-test with Welch’s correction was performed; **P<0.01, and ***P<0.001, adipocytes incubated with hATT-CM *vs.* Ctrol-CM; #P<0.05, adipocytes incubated with hATT-CM *vs.* hATN-CM. Ctrol-CM, control conditioned media; hATN-CM, conditioned media from human normal breast adipose tissue explants; hATT-CM, conditioned media from human breast cancer adipose tissue explants; PRDM16, proline rich domain containing 16; TBX1, TATA box 1; PGC1α, peroxisome proliferator activated receptor γ coactivator 1 α; TOMM20, the outer mitochondrial membrane member 20.

In addition, we measured protein expression of other beige/brown related markers: PGC1α, PRDM16 and TBX1, in adipocytes incubated with hATT- and hATN-CM for 24 and 72 h (**Figures 6A, B**). hATT-CM significantly increased PGC1α expression after 24 h of incubation compared to hATN-CM (**Figure 6A**) and after 72 h of incubation in comparison to hATN- and Ctrol-CM (**Figure 6B**). Although there was no significant increase in TBX1 expression level after 24 and 72 h of treatment with hATT-CM (**Figures 6A, B**, respectively), PRDM16 showed a tendency to increase following incubation with hATT-CM for 72 h (**Figure 6B**). We analyzed the expression of TOMM20, a mitochondrial outer membrane protein. TOMM20 expression significantly increased after 24 h of treatment with hATT-CM (**Figure 6A**). This result is in agreement with the increase of PGC1α, master regulator of mitochondrial biogenesis.

The results suggest that hATT-CM are able to induce the browning of white mature adipocytes, where PGC1α (**Figure 6B**), as well as PPARγ (**Figure 4B**) play a crucial role in the early browning process.

### Effects of soluble factors from hATT and hATN on Plin1, HSL and ATGL subcellular localization and protein expression levels in white adipocytes

**Figure 7:**
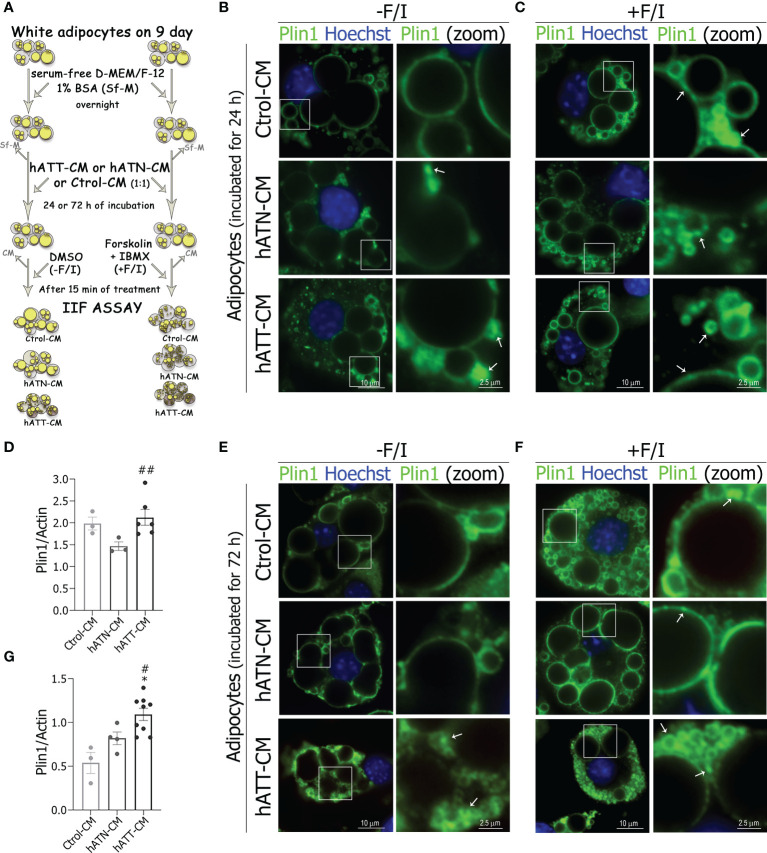
Effect of CM from hATN and hATT on the localization/protein expression of Plin1 in mature adipocytes. 3T3-L1 adipocytes were incubated with hATN-, hATT- or Ctrol-CM followed by incubation with DMSO (-F/I, basal condition) or 20 µM forskolin plus 0.5 mM IBMX (+F/I, lipolytic condition) for 15 min. **(A)** Schematic experimental approach. 3T3-L1 adipocytes after 24 **(B, C)** and 72 h **(E, F)** of treatment, under basal condition **(B, E)** or lipolytic condition **(C, F)**, were subjected to IIF with the indicated antibodies, and images were analyzed by confocal microscopy (magnification x600; see Materials and methods section for further details). Boxed areas are shown as zoomed images on right: Plin1 (zoom). White arrows indicate Plin1 segregation. Notably, the rimming observed on the pattern of Plin1 immunofluorescence on large LDs evidenced less fluorescence intensity in adipocytes treated with hATT-CM and hATN-CM for 24 and 72 h, under basal conditions. 3T3-L1 adipocytes were incubated with hATN-, hATT- or Ctrol-CM for 24 and 72 h, then lysed and Plin1 expression was measured by WB **(D, G)**. Actin was used as a loading control. (n = 3 experiments in duplicate). Images were analyzed by densitometry. Student’s t-test with Welch´s correction was performed; *P<0.05, adipocytes incubated with hATT-CM *vs.* Ctrol-CM; #P<0.05, and ##P<0.01, adipocytes incubated with hATT-CM *vs.* hATN-CM. Ctrol-CM, control conditioned media; hATN-CM, conditioned media from human normal breast adipose tissue explants; hATT-CM, conditioned media from human breast cancer adipose tissue explants; Plin1, perilipin 1.

In adipocytes, LDs size reflect the balance of TG synthesis (lipogenesis) and hydrolysis (lipolysis). Plin1, HSL and ATGL play a critical role in regulating lipid storage and release in the adipocyte (35–37). To determine whether lipolysis is involved in the morphological changes induced by hATT-CM in 3T3-L1 adipocytes, we analyzed the effect of different CM on subcellular localization and expression levels of each of these proteins. We evaluated lipolysis in adipocytes under basal (-F/I) and lipolytic (+F/I; 20 µM forskolin plus 0.5 mM IBMX) conditions, (**Figure 7A**, schematic approach) because the patterns of subcellular distribution of these lipolytic proteins is a cAMP-dependent event.

Under basal conditions, adipocytes incubated for 24 h with hATT- and hATN-CM showed a strong signal for Plin1, clearly located on the surface of small-LDs, and to a lesser extent on large-LD when compared to Ctrol-CM (**Figure 7B**, *bottom* and *middle panels vs. top panels*). Notably, hATT-CM, and to a lesser extent hATN-CM, induced accumulation of Plin1 in distinct micro domains on small-LDs, thus suggesting a possible segregation of Plin1, mainly in peripheral small and micro-LDs (**Figure 7B**, *bottom* and *middle right panels*, respectively). This localization pattern of Plin1 was similar to that observed in adipocytes incubated with Ctrol-CM under lipolytic conditions (**Figure 7B**, *bottom* and *middle panels vs.* **Figure 7C** *top right panel*). Moreover, hATT-CM increased the protein expression level of Plin1 after 24 h of treatment with respect to hATN-CM, under basal conditions (**Figure 7D**). The effect of hATT-CM on Plin1 relocalization was greater after 72 h of treatment (**Figure 7E, F**, *bottom panels vs.* B and C *bottom panels*). In fact, hATT-CM promoted the relocalization of Plin1 on the LDs prior to lipolytic stimulation (**Figure 7E**, *bottom panels*). As expected, the effect of hATT-CM on the segregation of Plin1 and the increase in the number of micro-LDs was exacerbated under lipolytic conditions (**Figure 7F**, *bottom panels vs.* E *bottom panels*). hATT-CM significant increased Plin1 protein expression after 72 h of incubation compared to hATN- and Ctrol-CM (**Figure 7G**).

**Figure 8:**
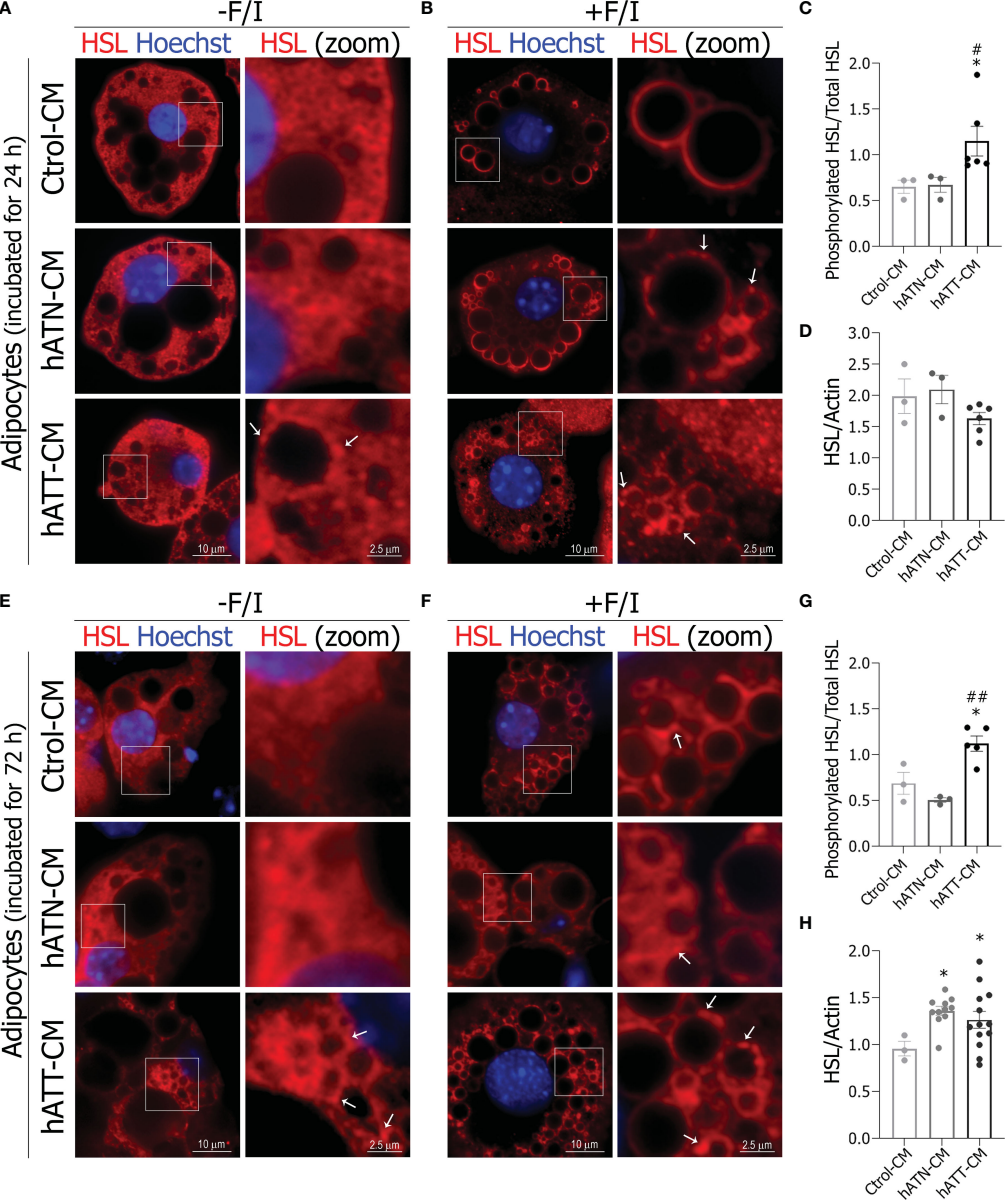
Effect of CM from hATN and hATT on localization/protein expression of HSL in mature adipocytes. 3T3-L1 adipocytes were incubated with hATN-, hATT- or Ctrol-CM for 24 h **(A, B)** and 72 h **(E, F)** followed by incubation with DMSO (-F/I, basal condition) or 20 µM forskolin plus 0.5 mM IBMX (+F/I, lipolytic condition) for 15 min. Then, 3T3-L1 adipocytes were subjected to IIF with the indicated antibodies, and images were analyzed by confocal microscopy (magnification x600; see Materials and methods section for further details). Boxed areas are shown as zoomed images on right: HSL (zoom). White arrows indicate domains of HSL localized on LDs. 3T3-L1 adipocytes were incubated with hATN-, hATT- or Ctrol-CM for 24 **(C, D)** and 72 h **(G, H)**, and then lysed. The Ser660 phosphorylated HSL and total HSL expression levels were analyzed by WB. Actin was used as a loading control. Images were analyzed by densitometry. (n = 3 experiments in duplicate). Student’s t-test with Welch´s correction was performed; *P<0.05, adipocytes incubated with hATT-CM or hATN-CM *vs.* Ctrol-CM; #P<0.05, and ##P<0.01, adipocytes incubated with hATT-CM *vs.* hATN-CM. Ctrol-CM, control conditioned media; hATN-CM, conditioned media from human normal breast adipose tissue explants; hATT-CM, conditioned media from human breast cancer adipose tissue explants; HSL, hormone sensitive lipase.

The process of lipolysis is mainly regulated by the spatial recruitment of lipases, HSL and ATGL from the cytoplasm to the LDs surface. Under basal conditions, hATT-CM induced a partially relocation of HSL from cytoplasm to small domains on LDs surface after 24 h of incubation in comparison to hATN- and Ctrol-CM (**Figure 8A**, *bottom right panel vs. middle* and *top right panels*). Under lipolytic conditions, hATN- and hATT-CM promoted the complete translocation of HSL from a cytoplasmic localization to LDs surface, a critical step in the lipolytic process (**Figure 8B**). Also, the effect of hATT-CM on HSL relocalization on LD was exacerbated and hATN-CM showed a lesser effect than Ctrol-CM (*Note white arrows* **Figure 8B**, *bottom* and *middle right panels vs. top right panel*). Because the phosphorylation of HSL in Ser-660 is required to activate lipolysis and HSL translocation to the LDs, we analyzed the ability of hATT-CM to induce phosphorylation of HSL after 24 h of incubation, under basal conditions. hATT-CM significantly increased phosphorylation of HSL Ser-660 with respect to hATN- and Ctrol-CM (**Figure 8C**). No significant differences in HSL protein expression were observed in adipocytes incubated with the different CM studied after 24 h (**Figure 8D**). The effect of hATT-CM on HSL translocation on the LDs surface was greater after 72 h of treatment, under basal and lipolytic conditions (**Figures 8E, F** *bottom panels vs.* A and B *bottom panel*s). Notably, hATT-CM induced a complete translocation of HSL from a cytoplasmic localization to LDs surface after 72 h of treatment under basal conditions (**Figure 8E** *bottom vs. top panels*). hATT-CM significantly increased phosphorylation of HSL Ser-660, compared to hATN- and Ctrol-CM after 72 h of incubation, under basal conditions (**Figure 8G**). This effect was also observed after 120 h of incubation with hATT-CM (**Additional file: Supplementary Figure S2**). hATT- and hATN-CM significantly increased HSL protein expression level in comparison to Ctrol-CM after 72 h of incubation (**Figure 8H**).

**Figure 9:**
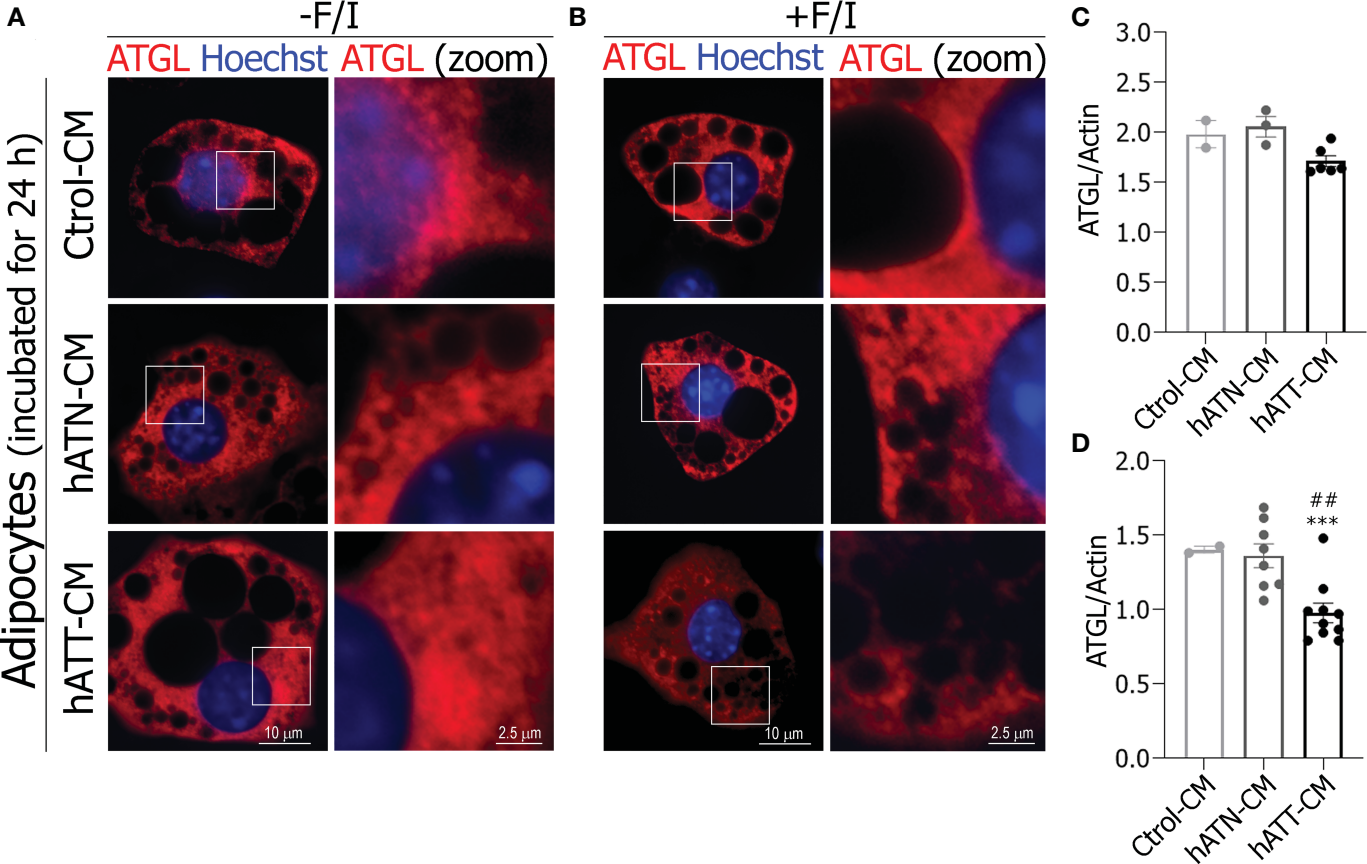
Effect of CM from hATN and hATT on localization/expression of ATGL in mature adipocytes. 3T3-L1 adipocytes were incubated with hATN-, hATT- or Ctrol-CM for 24 h followed by incubation with DMSO (**A**, -F/I, basal condition) or 20 µM forskolin plus 0.5 mM IBMX (**B**, +F/I, lipolytic condition) for 15 min. Then, adipocytes were subjected to IIF with the indicated antibodies, and images were analyzed by confocal microscopy (magnification x600; see Materials and methods section for further details). Boxed areas are shown as zoomed images on right: ATGL (zoom) (n = 3 experiments in duplicate). 3T3-L1 adipocytes were incubated with hATN-, hATT- or Ctrol-CM for 24 **(C)** and 72 h **(D)**, and then lysed. ATGL expression level was measured by WB. Actin was used as a loading control. Images were analyzed by densitometry. (n = 2 experiments in duplicate with similar results). Student’s t-test with Welch´s correction was performed; ***P<0.001, adipocytes incubated with hATT-CM *vs.* Ctrol-CM. ##P<0.01, adipocytes incubated with hATT-CM *vs.* hATN-CM. Ctrol-CM, control conditioned media; hATN-CM, conditioned media from human normal breast adipose tissue explants; hATT-CM, conditioned media from human breast cancer adipose tissue explants; ATGL, adipose triglyceride lipase.

In adipocytes incubated with hATT- and hATN-CM for 24 h, no appreciable differences were found in the ATGL distribution pattern with respect to Ctrol-CM, where ATGL was diffusely distributed throughout the cytoplasm and near to the nucleus (**Figure 9A** *bottom and middle panels* vs *top panel*s). As was observed for HSL, hATN- and hATT-CM promoted the translocation of ATGL from a cytoplasmic localization to LDs surface under lipolytic conditions (**Figure 9B**, *bottom* and *middle panels* vs *top panels*). Interestingly, hATT-CM promoted the almost complete translocation of ATGL from cytoplasmic to around LDs, in comparison to hATN- and Ctrol-CM (**Figure 9B**, *bottom right panel* vs. *middle* and *top right panels*). Also, we observed that hATT-CM decreased ATGL expression after 24 h of incubation, with differences being significant after 72 h of incubation (**Figures 9C, D**, respectively).

### Effects of soluble factors from hATT and hATN on intracellular signal pathways in mature adipocytes

**Figure 10:**
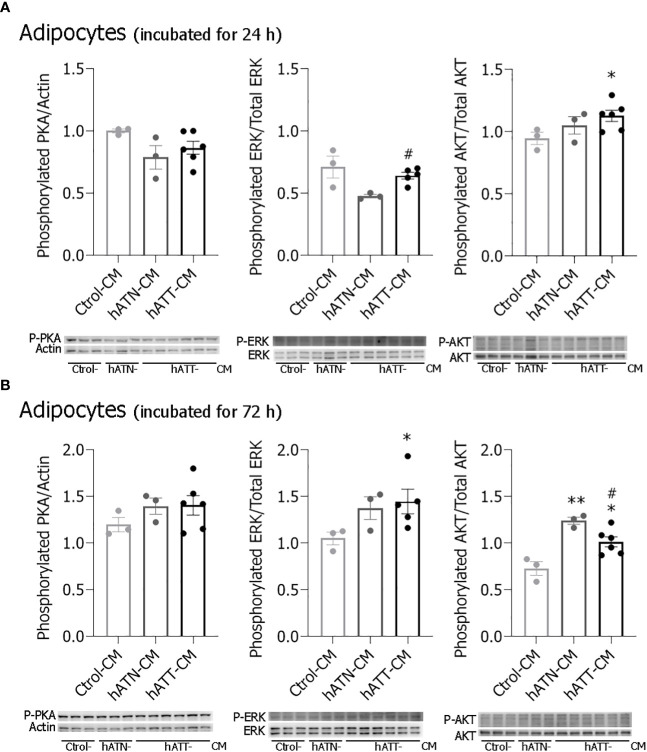
Effects of factors secreted by peritumoral adipocytes on different signaling pathways of mature 3T3-L1 adipocytes. 3T3-L1 adipocytes were incubated with hATN-, hATT- or Ctrol-CM for 24 **(A)** and 72 h **(B)**, and then lysed. Expression of Thr198 phosphorylated PKA, Tyr204 phosphorylated ERK, total ERK, Thr308 phosphorylated AKT and total AKT were measured by WB. Actin was used as a loading control. Images were analyzed by densitometry. Student’s t-test with Welch´s correction was performed; *P< 0.05, and **P< 0.01, adipocytes incubated with hATT-CMs or hATN-CM *vs.* Ctrol-CM; #P< 0.05, adipocytes incubated with hATT-CM *vs*. hATN-CM. Ctrol-CM, control conditioned media; hATN-CM, conditioned media from human normal breast adipose tissue explants; hATT-CM, conditioned media from human breast cancer adipose tissue explants; PKA, protein kinase A; ERK, extracellular signal regulated kinase; AKT, protein kinase B.

In conjunction PKA, ERK and AKT have been demonstrated to play an important role in lipolysis (38). To elucidate the intracellular signal pathways involved in the observed changes, pPKA, pERK and pAKT expression were evaluated in adipocytes incubated for 24 and 72 h with different CM. hATT-CM significantly increased pAKT expression level in adipocytes incubated for 24 h compared to Ctrol- and hATN-CM (**Figure 10A**). No significant differences in pPKA and pERK expression levels were observed after short-term treatment. Although pPKA expression level showed no significant differences, a tendency to increase was seen after 72 h of incubation with hATT-CM (**Figure 10B**). Furthermore, hATT-CM significantly increased pERK expression compared to Ctrol- and hATN-CM (**Figure 10B**). hATN- and hATT-CM significantly increased pAKT expression with respect to Ctrol-CM in adipocytes after 72 h of incubation (**Figure 10B**). After 120 h of treatment, hATT-CM significant increased pAKT (**Additional file: Supplementary Figure S3**).

## Discussion

We have showed that peritumoral AT secrete factors into the medium that regulates the behavior of breast cancer cells (e.g., proliferation, adhesion and migration) (13, 32). In this paper, we found that hATT-CM induced a morphological transition in white adipocytes by paracrine action decreasing adipocyte cell size, increasing the number of small and micro-LDs per cell and the endogenous TG content. These effects increased after medium and long-term treatment, suggesting that soluble factors from hATT could have a cumulative effect that leads to morphological changes such as the increase of small and micro-LDs and that they can trigger certain processes, thus altering the metabolic function of the adipocytes. Recent studies have associated the trans-differentiation adipocytes (white to beige) with breast cancer. Browning of mammary fat has been reported close to malignant tumors compared to the vicinity of benign breast lesions suggesting that browning is directly induced by tumor (17, 18, 20, 29). Also, adipocytes from the tumor microenvironment show phenotypical alterations and occurrence of an activated phenotype. The delipidation of peritumoral adipocytes is the result of at least 2 processes, including “dedifferentiation” as a consequence of the activation of the Wnt/β-catenin pathway, concomitantly with the increase of the expression of Pref-1 and lipolysis (14, 39, 40). To elucidate whether these two processes occur in our study model, we evaluated the ability of hATT-CM to modulate the expression of key players of adipogenesis regulation and the maintenance of a differentiated state in adipocytes by paracrine/autocrine action.

We demonstrated that hATT- and hATN-CM have the ability to modulate adipogenic and mature adipose markers expression in a time-dependent manner. While hATN-CM increased PPARγ and FABP4 expression after the short- and medium-term incubation of adipocytes, respectively, hATT-CM increased C/EBPβ LIP/LAP ratio and decreased FABP4 and PPARγ protein expression after a long-term treatment. These results suggest that soluble factors from hATN are in favor of maintaining a mature adipose state, while hATT-CM would promote a less differentiated state after long-term treatment. Another evidence of this latter action was the increased level of Pref-1 and concomitantly increased pERK after treatment with hATT-CM. However, the expression level of Pref-1 decreased in adipocytes incubated with hATT-CM after long-term treatment. We speculate, in agreement with previous reports (41, 42), that Pref-1 could be cleaved by soluble factors present in hATT-CM to generate a soluble form released by adipocytes to act as an autocrine/paracrine factor *via* MEK/ERK (41, 42). Despite the fact that Pref-1 increased in adipocytes treated with hATN-CM, there were no significant differences in C/EBPβ-LAP and FABP4 expression levels. Notably, the increase of Pref-1 induced by hATN-CM, had no significant effect on ERK activation in mature white adipocytes. These results suggest that Pref-1 could play another role beyond an adipogenic inhibitory function. Future studies will be needed to deepen these findings.

Caveolae play important roles in the regulation of cell signaling and metabolism. In fact, the loss of the expression of stromal CAV-1 is a functional marker of autophagy, oxidative stress, and hypoxia in the tumor microenvironment, favoring tumor progression (43, 44). Importantly, loss of stromal CAV-1 is associated with poor patient outcomes in breast cancer (44). In concordance, an increase in C/EBPβ-LIP isoform expression supports a pro-tumorigenic microenvironment and plays a role in autophagy, resistance to apoptosis, and changes in cytokine/chemokine expression (45, 46). The present results revealed that hATT-CM decreased CAV-1 and increased C/EBPβ-LIP protein expression in adipocytes, further supporting the contention that the breast cancer peritumoral AT could contribute to tumor progression. Therefore, adipocytes present at the invasive front release soluble factors that favor a progressive adjacent white adipocyte atrophy by autocrine/paracrine action. However, our results suggest that before achieving a “dedifferentiated” state, adipocytes undergo a lipid metabolic imbalance, given that we observed that browning and lipolysis were induced in white adipocytes by soluble factors released from hATT.

WAT browning is triggered by the increased gene expression of different brown/beige adipogenic markers (47, 48). Up-regulation of UCP1 is very robust evidence of the browning of white adipocytes, since respiratory uncoupling is important to the distinctive brown/beige AT phenotype of (49). Also, overexpression of PGC1α, a master regulator of mitochondrial biogenesis, stimulates the expression of UCP1 and other essential mitochondrial proteins of the respiratory chain in white adipocytes. We observed that UCP1, PCG1α, and PPARγ expression levels were significantly higher in adipocytes treated with hATT-CM rather than hATN-CM when compared to Ctrol-CM, suggesting a shift in adipocyte phenotype (white to beige). The browning of the white adipocytes includes marked changes in the mitochondrial biogenesis and metabolic state of mitochondria (50). Accordingly, our present results show that an increased expression of PCG1α and TOMM20 occurs in adipocytes treated with hATT-CM, supporting mitochondrial biogenesis. In addition, UCP1-positive mitochondria preferentially displayed a round shape (mitochondrial fission), suggesting that hATT-CM could induce a metabolic mitochondrial switch. These evidences are in line with previous reports showing an increased mitochondrial fission activity is associated with high tumor progression in some cancer cells. Also, the expression of PGC1α is altered in tumors and metastasis in relation to modifications in cellular metabolism (51, 52). Our results further support the contention that soluble factors released by hATT could contribute to white adipocyte browning observed in the tumor microenvironment and promote a mitochondrial switch in both microenvironment and breast cancer cells in favor of tumor progression, since both processes play a role in breast tumor development (31, 53).

Previously, Sawada et al. demonstrated that overexpression of Plin1 in 3T3-L1 adipocytes induces a brown AT-like phenotype, beyond a decrease in LD size (54). In agreement, we observed that adipocytes treated with hATT-CM increase Plin1 protein expression level, which not only could induce browning, but also regulate LD remodeling.

Plin1 functions as a ‘‘barrier’’ between stored neutral lipid and lipases and together with HSL are important regulators of LD turnover (38, 55, 56). In this study, Plin1 and HSL expression levels were increased in adipocytes treated with hATT-CM. Also, hATT-CM had the ability to modulate the subcellular localization of lipolytic proteins, favoring HSL translocation (cytoplasm to the LD surface) and Plin1 reorganization on LDs, under basal conditions. In fact, both proteins are restricted to a subpopulation of LDs, and it was suggested that micro-LDs may represent an active pool of LDs undergoing active lipolysis (57).

Our results showed that the soluble factors present in hATT-CM induce an activated phenotype in mature adipocytes which promotes TG lipolysis mainly in micro-LDs. Thus, LD turnover is accompanied by a significant remodeling of LDs, as was observed in adipocytes after treatment with hATT-CM. However, we did not observe a significant increase in glycerol release after 24 h of treatment, despite a decrease in TG content. This lack of an effect on glycerol release may be associated with the increased Plin1 expression, protecting LDs against their hydrolysis by lipases. There is evidence suggesting that ATGL regulates LD size and basal lipolysis independent of Plin1 expression (58). We observed that hATT-CM induced a decrease in protein expression of ATGL in adipocytes without affecting its subcellular localization, suggesting that HSL together with Plin1 would be responsible for the remodeling of LDs. Additionally, hATT-CM induced HSL activation, as evidenced by the increase of pHSL (Ser660) in adipocytes after short-, medium- and long-term treatment. These results suggest that soluble factors from hATT could induce TG hydrolysis by a canonical lipolysis pathway. We did not discard the possibility that PKA activation occurs, even though no significant difference was observed in pPKA. Activation of lipolysis through phosphorylation of HSL by ERK1/2 could also be occurring. ERK1/2 regulate adipocyte lipolysis in response to mitogens, growth factors or cytokine signals by phosphorylating HSL on Ser600 and promoting the activity of HSL (38). ERK could also be responsible for the browning observed in adipocytes incubated with hATT-CM. A previous study showed that ERK phosphorylates PPARγ at Ser273 as well as Ser112, inhibiting its activity. The inhibition of PPARγ phosphorylation on Ser273 increases UCP1 expression in adipocytes (59). Thus, the increase in pERK not only induces an increase in HSL activation and concomitantly lipolysis stimulation, but also could promote PPARγ activity and thus modulate white adipocyte browning. Regarding our results, hATT-CM significantly increased pERK in adipocytes with respect to hATN- and Ctrol-CM after medium-term treatment. Thus, lipolysis and browning induced by hATT-CM would be regulated by pERK. In relation to AKT, recent studies have shown that the phosphorylated form is involved in adipocyte browning, a pathway regulated by insulin (60). Thus, soluble factors released by peritumoral adipocytes could also induce paracrine browning of adipocytes *via* AKT and ERK phosphorylation, because hATT-CM induced an increase in pAKT and pERK in mature adipocytes. Lines of evidence suggest that there is a correlation between pAKT and mitochondrial fragmentation (61). In fact, hATT-CM could induce a metabolic mitochondrial switch through the pAKT pathway, in agreement to above mentioned. Additionally, we will analyze the effect of soluble factors released by peritumor adipocyte on alteration of biogenesis and metabolic mitochondrial switch. These experiments are in progress and will be published as a continuation of this study.

Taken together, the results presented in this paper provide significant evidence that peritumoral AT adipocytes, activated by the presence of tumor cells, secrete factors into the medium that could change their microenvironment by paracrine action on adjacent adipocytes, inducing browning and lipolysis and possibly a “dedifferentiated” given that the long-term treatment led to a decrease in the expression of FABP4, PPARγ, and to a lesser extent CAV-1, while the C/EBPβ-LIP isoform, which is known to act as a trans-dominant repressor, increased in adipocytes undergoing morphological remodeling. Despite these findings, additional investigation is necessary to fully understand this phenomenon.

We conclude that adipocytes from the tumor microenvironment exhibit an activated phenotype that could have been induced not only by secreted soluble factors from epithelial cells but also by paracrine action from other adipocytes present in this microenvironment, thus suggesting a “domino effect”. We propose that browning, lipolysis and possibly the “dedifferentiated” state of adipocytes attached to the tumor could be considered as prognostic factors in breast cancer. Further research is needed to identify the relationship between these processes and tumor progression, that could eventually lead to the development of new ways for cancer treatment. We think that these findings could probably be extended to other types of tumors, depending on the composition of the tumor microenvironment.

## Data availability statement

The original contributions presented in the study are included in the article/**Supplementary Material**. Further inquiries can be directed to the corresponding author.

## Ethics statement

The studies involving human participants were reviewed and approved by IBYME (CE 025) and the Churruca-Visca Police Medical Centre IRBs. The patients/participants provided their written informed consent to participate in this study.

## Author contributions

PP performed part of the experiments, analyzed data, interpreted results, drafted manuscript, edited, revised manuscript. MG performed part of the experiments, helped draft the manuscript. AL analyzed data, contributed to the discussion. SF, ML, NS, AU, CF, AA, and RD contributed with part of the biological samples used, drafted the manuscript. JC conceived the study, drafted, edited and revised the manuscript. JT conceived and designed research and performed the research, analyzed data, interpreted results of experiments, prepared figures, draft manuscript, edited and revised manuscript. All authors contributed to the article and approved the submitted version.
