# Supplementary material for: Peritumoral adipose tissue promotes lipolysis and white adipocytes browning by paracrine action
Source: Front Endocrinol (Lausanne). 2023 Apr 26;14:1144016. doi: 10.3389/fendo.2023.1144016 (PMC10170974; doi:10.3389/fendo.2023.1144016)
Supplement: Supplementary file 1 [file DataSheet_1.docx]

## Supplementary Table and Figure legends

## Table S1

Antibody information and dilutions used to detect subcellular localization using IIF.

| **Antibody** | **Catalogue** | **Dilution** |
| --- | --- | --- |
| Alexa Fluor 555 | ab150074 | 1:400 |
| ATGL | ab240381 | 1:500 |
| FITC 488 | F0382 | 1:100 |
| Hoechst | 33342 | 1:1000 |
| HSL | ab45422 | 1:200 |
| Lipid-TOX | HCS Lipid‑TOX™ Neutral lipid Stains | 1:300 |
| Perilipin (Plin1) | ab3526 | 1:500 |
| UCP1 | ab10983 | 1:500 |
| WGA, Wheat germ agglutinin | WGA, Alexa Fluor^®^ 488 conjugate | 1:1000 |

## Table S2

Antibody information and dilutions used to detect protein expression using WB assay.

| **Antibody** | **Catalogue** | **Dilution** |
| --- | --- | --- |
| Actin | I19 sc-1616R | 1:1000 |
| AKT | sc-8312 | 1:1000 |
| ATGL | ab240381 | 1:1000 |
| C/EBPβ | ab32358 | 1:1000 |
| CAV-1 | 610060 BD | 1:1000 |
| ERK | sc-94 | 1:1000 |
| FABP4 | ab219595 | 1:2000 |
| HSL | ab45422 | 1:1000 |
| pAKT (Thr308) | sc-16646 | 1:500 |
| pERK (Tyr204) | sc-7383 | 1:1000 |
| PGC1α | ab54481 | 1:1000 |
| pHSL (Ser660) | Cell Signaling 4126 | 1:1000 |
| Perilipin (Plin1) | ab3526 | 1:2000 |
| PPARγ | E8 sc-7273 | 1:500 |
| pPKA (Thr198) | sc-32968 | 1:1000 |
| PRDM16 | ab106410 | 1:1000 |
| Pref-1 (DLK-1) | ab119930 | 1:1000 |
| TBX1 | ab18530 | 1:500 |
| TOMM20 | ab186734 | 1:1000 |
| UCP1 | U6382 | 1:1000 |
| UCP1 | ab10983 | 1:1000 |

## Figure S1

**
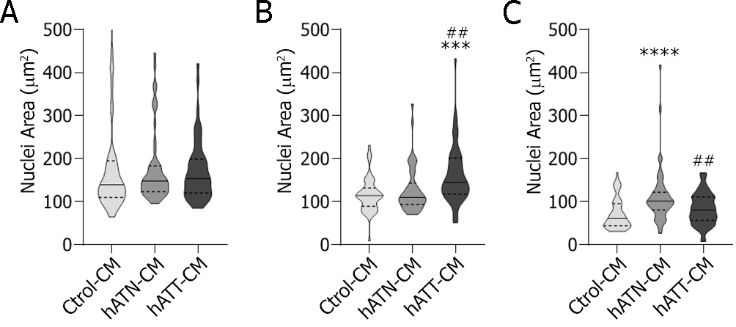
**

**Effect of CM from hATN and hATT on nucleus size in adipocytes**

3T3-L1 adipocytes were incubated with hATN-, hATT- or Ctrol-CM for 24, 72 and 120 h, subjected to IIF and images were analyzed by confocal microscopy. Nuclei size (μm^2^) were assessed from 64 adipocytes after incubation with the different CM for 24 (A), 72 (B) and 120 h (C) by the Hoechst fluorescence. Data presented as mean ± SEM of three independent experiments. Tukey's multiple comparison test was performed; ***P<0.001, and ****P<0.0001 hATT-CM or hATN-CM *vs.* Ctrol-CM; ##P<0.01, hATT-CM *vs.* hATN-CM. Ctrol-CM, control conditioned media; hATN-CM, conditioned media from human normal breast adipose tissue explants; hATT-CM, conditioned media from human breast cancer adipose tissue explants.

## Figure S2

**
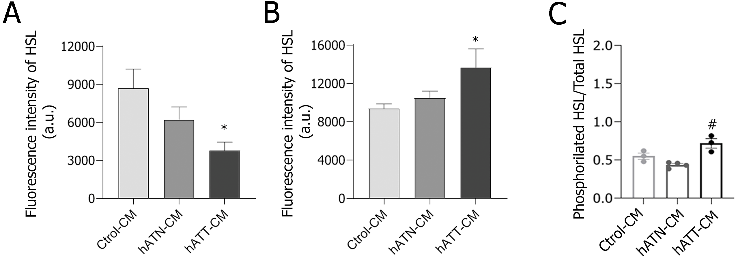
**

**Effect of CM from hATN and hATT on HSL fluorescence intensity and Ser660 phosphorylation** **in adipocytes**

3T3-L1 adipocytes were incubated with hATN-, hATT- or Ctrol-CM for 24 and 72 h, subjected to IIF with the indicated antibodies, and images were analyzed by confocal microscopy. Fluorescence intensity of HSL were measured from 50 adipocytes incubated with the different CM for 24 (A) and 72 h (B). Data presented as mean ± SEM of two independent experiments. Tukey's multiple comparison test was performed. 3T3-L1 adipocytes were incubated with hATN-, hATT- or Ctrol-CM for 120 h and then lysed. Expression of Ser660 phosphorylated HSL and total HSL were measured by Western blot (C). Images were analyzed by densitometry. Data presented as mean ± SEM of two independent experiments. Student's t-test with Welch's correction was performed; *P<0.05 adipocytes incubated with hATT-CM *vs.* Ctrol-CM. #P<0.05, adipocytes incubated with hATT-CM *vs.* hATN-CM. a.u., arbitrary units; Ctrol-CM, control conditioned media; hATN-CM, conditioned media from human normal breast adipose tissue explants; hATT-CM, conditioned media from human breast cancer adipose tissue explants; HSL; hormone sensitive lipase.

## Figure S3

**
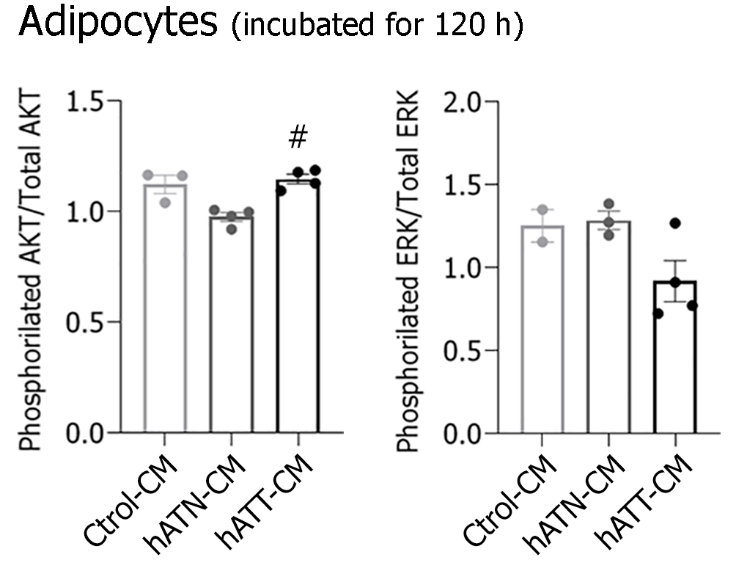
**

**Effect of CM from hATN and hATT on intracellular signal pathways in adipocytes after long-term treatment**

3T3-L1 adipocytes were incubated with hATN-, hATT- or Ctrol-CM for 120 h and then lysed. Expression of Thr308 phosphorylated AKT, total AKT, Tyr204 phosphorylated ERK, and total ERK were measured by Western blot. Images were analyzed by densitometry. Data presented as mean ± SEM of two independent experiments. Student's t-test with Welch´s correction was performed; #P<0.05, adipocytes incubated with hATT-CM *vs.* hATN-CM. Ctrol-CM, control conditioned media; hATN-CM, conditioned media from human normal breast adipose tissue explants; hATT-CM, conditioned media from human breast cancer adipose tissue explants; HSL, hormone sensitive lipase; AKT, protein kinase B; ERK, extracellular signal regulated kinase.
